# Complete genome sequence of the phenanthrene-degrading soil bacterium *Delftia acidovorans* Cs1-4

**DOI:** 10.1186/s40793-015-0041-x

**Published:** 2015-08-15

**Authors:** Ameesha R. Shetty, Vidya de Gannes, Chioma C. Obi, Susan Lucas, Alla Lapidus, Jan-Fang Cheng, Lynne A. Goodwin, Samuel Pitluck, Linda Peters, Natalia Mikhailova, Hazuki Teshima, Cliff Han, Roxanne Tapia, Miriam Land, Loren J. Hauser, Nikos Kyrpides, Natalia Ivanova, Ioanna Pagani, Patrick S. G. Chain, Vincent J Denef, Tanya Woyke, William J. Hickey

**Affiliations:** O.N. Allen Laboratory for Soil Microbiology, Department of Soil Science, University of Wisconsin-Madison, Madison, WI 53706 USA; Department of Food Production, University of the West Indies, St. Augustine, Trinidad and Tobago; Department of Microbiology, University of Lagos, Lagos, Nigeria; DOE Joint Genome Institute, Walnut Creek, CA USA; Algorithmic Biology Lab, St. Petersburg Academic University, St.Petersburg, Russia; Bioscience Division, Los Alamos National Laboratory, Los Alamos, NM USA; Oak Ridge National Laboratory, Oak Ridge, TN USA; Department of Ecology and Evolutionary Biology, University of Michigan, Ann Arbor, MI USA

**Keywords:** *Delftia acidovorans* Cs1-4, Genome, *phn* island, Phenanthrene, polycyclic aromatic hydrocarbons, Nanopods

## Abstract

Polycyclic aromatic hydrocarbons (PAH) are ubiquitous environmental pollutants and microbial biodegradation is an important means of remediation of PAH-contaminated soil. *Delftia acidovorans* Cs1-4 (formerly *Delftia* sp. Cs1-4) was isolated by using phenanthrene as the sole carbon source from PAH contaminated soil in Wisconsin. Its full genome sequence was determined to gain insights into a mechanisms underlying biodegradation of PAH. Three genomic libraries were constructed and sequenced: an Illumina GAii shotgun library (916,416,493 reads), a 454 Titanium standard library (770,171 reads) and one paired-end 454 library (average insert size of 8 kb, 508,092 reads). The initial assembly contained 40 contigs in two scaffolds. The 454 Titanium standard data and the 454 paired end data were assembled together and the consensus sequences were computationally shredded into 2 kb overlapping shreds. Illumina sequencing data was assembled, and the consensus sequence was computationally shredded into 1.5 kb overlapping shreds. Gaps between contigs were closed by editing in Consed, by PCR and by Bubble PCR primer walks. A total of 182 additional reactions were needed to close gaps and to raise the quality of the finished sequence. The final assembly is based on 253.3 Mb of 454 draft data (averaging 38.4 X coverage) and 590.2 Mb of Illumina draft data (averaging 89.4 X coverage). The genome of strain Cs1-4 consists of a single circular chromosome of 6,685,842 bp (66.7 %G+C) containing 6,028 predicted genes; 5,931 of these genes were protein-encoding and 4,425 gene products were assigned to a putative function. Genes encoding phenanthrene degradation were localized to a 232 kb genomic island (termed the *phn* island), which contained near its 3’ end a bacteriophage P4-like integrase, an enzyme often associated with chromosomal integration of mobile genetic elements. Other biodegradation pathways reconstructed from the genome sequence included: benzoate (by the acetyl-CoA pathway), styrene, nicotinic acid (by the maleamate pathway) and the pesticides Dicamba and Fenitrothion. Determination of the complete genome sequence of *D. acidovorans* Cs1-4 has provided new insights the microbial mechanisms of PAH biodegradation that may shape the process in the environment.

## Introduction

**Fig. 1:**
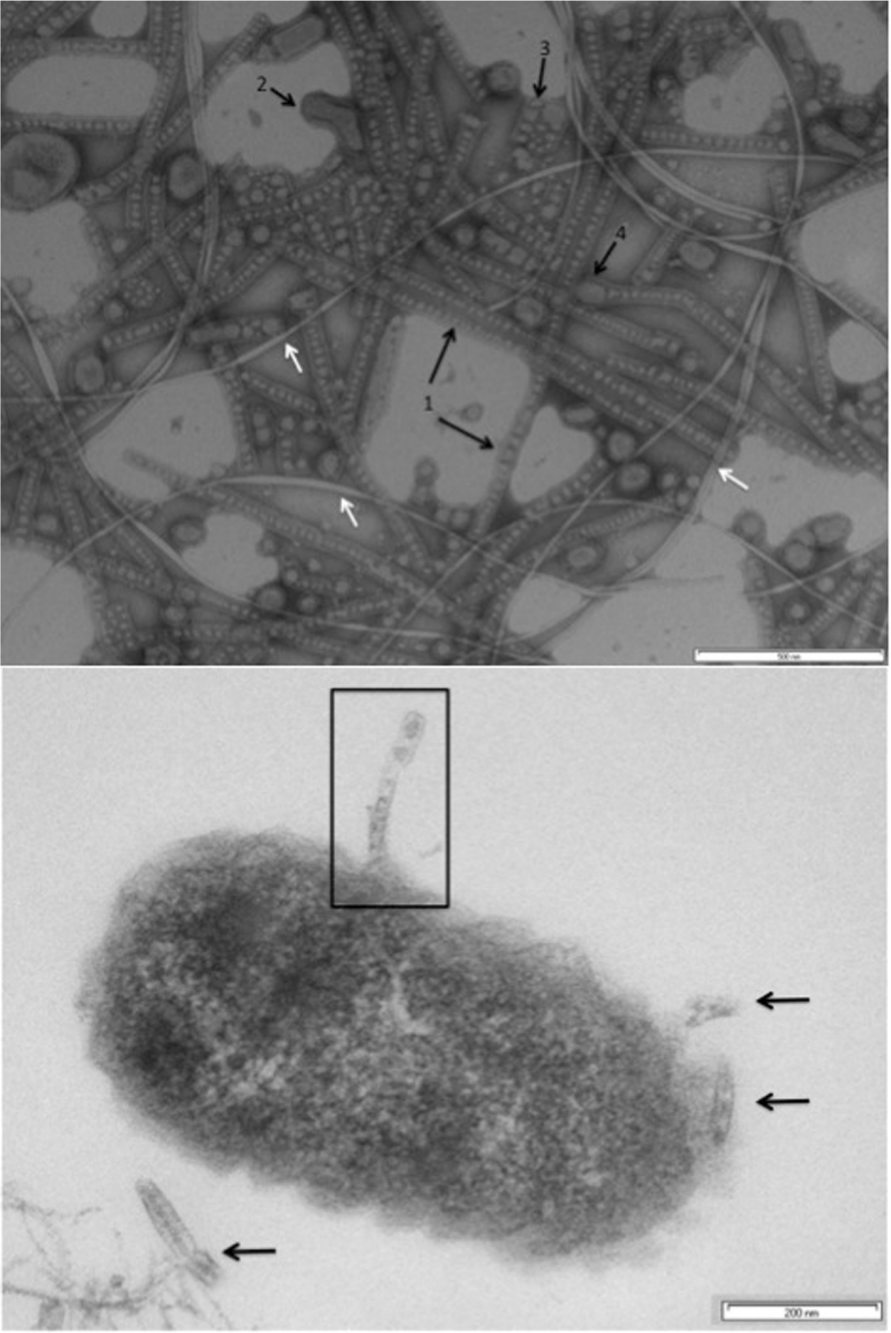
Top panel: Transmission electron micrograph of negatively-stained culture fluids from phenanthrene-grown *D. acidovorans* Cs1-4. Nanopods and related structures are indicated by the black arrows, flagella are shown by white arrows. Features indicated by numbers are: 1.) linear structures, 2.) globular elements, 3.) blocky formations and 4.) linear structure with globular element at terminus. The scale bar at bottom right represents 500 nm. Bottom panel: Thin section transmission electron micrograph of *D. acidovorans* Cs1-4 cell grown as a biofilm on phenanthrene. A nanopod growing from the cell surface is enclosed by the box and arrows indicate segments of other nanopods in the biofilm matrix. The scale bar at bottom right represents 200 nm

**Fig. 2:**
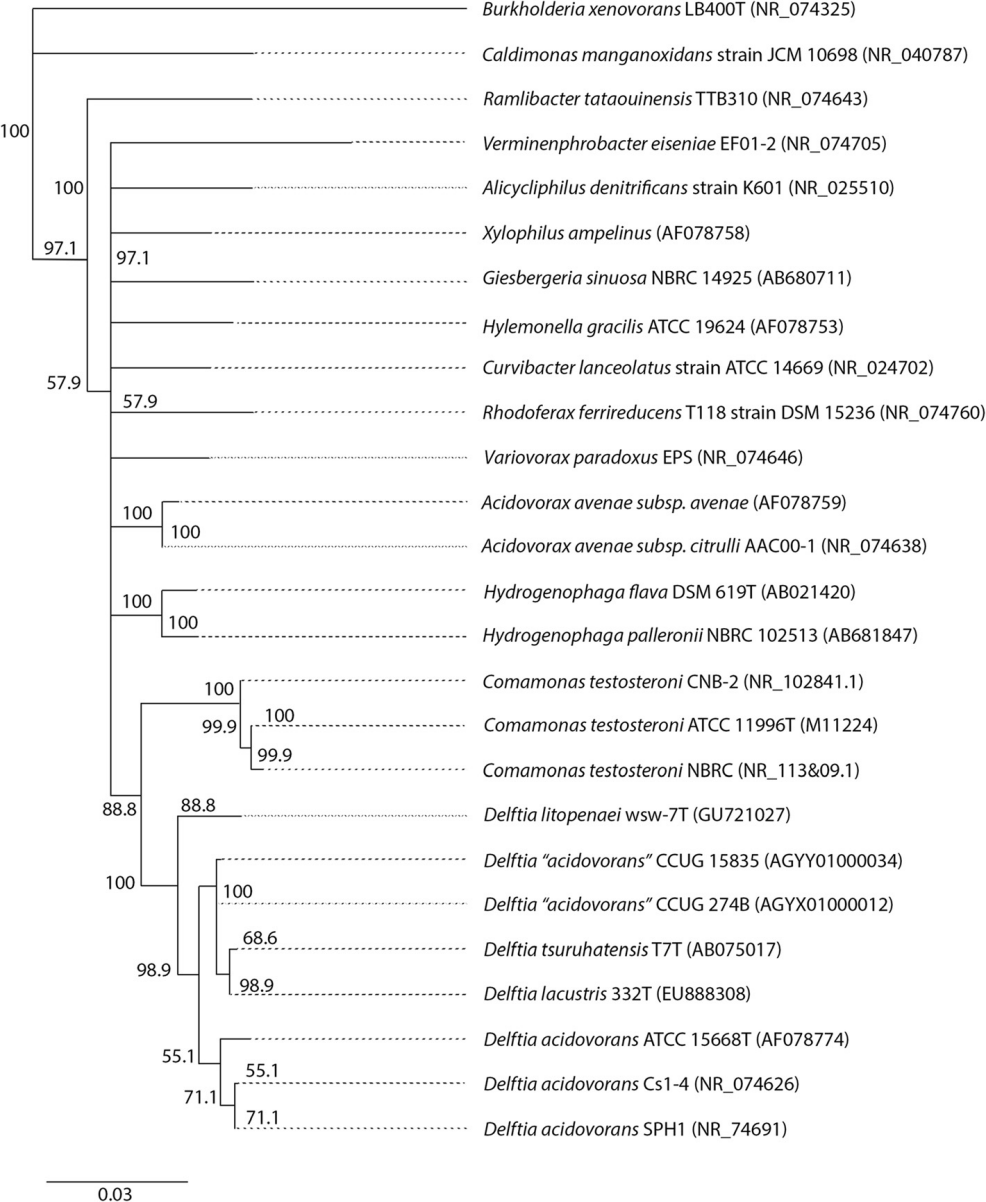
Phylogenetic tree highlighting the position of *Delftia acidovorans* Cs1-4 relative to other *Delftia* species as well as genera within the family *Comamonadaceae.* The 16S rRNA gene sequences were obtained from either type strains (strain designation ends with “T”) and/or have full genome sequence data that is publicly available (GenBank accession numbers are indicated in parentheses) were aligned by using MUSCLE (http://www.drive5.com/muscle/). MEGA *v.* 5.05 was used for phylogenetic reconstruction via the maximum-likelihood method (tree inference by nearest neighbor interchange), with the Tamura-Nei substitution model. Uniform bootstrap values obtained from 1000 replicate analyses are indicated at nodes. *Burkholderia xenovorans* LB400 (family *Burkholderiaceae*) was used as the outgroup. The scale bar represents a 0.03 % nucleotide sequence divergence

Polycyclic aromatic hydrocarbons (PAH) are ubiquitous environmental pollutants and microbial biodegradation is an important means of remediation of PAH-contaminated soil. *Delftia acidovorans* Cs1-4 (formerly *Delftia* sp. Cs1-4) was isolated using phenanthrene as the sole carbon source from PAH contaminated soil in Wisconsin [1] and its genome sequence was determined to gain insights into the mechanisms and pathways of PAH metabolism. *D. acidovorans* Cs1-4 was also unique as the strain in which the novel extracellular structures called nanopods were discovered [2]. Nanopods are extensions of the cell that consist of a surface layer protein encasing outer membrane vesicles (Fig. 1; [2]). The specific functions of nanopods in *D. acidovorans* Cs1-4 are unknown. But, some connection to phenanthrene degradation is likely as growth on that compound induces production of these structures [2] and mutants disabled in production of nanopods are impaired in growth on phenanthrene [2, 3].

Bacterial degraders of PAH can be grouped based on the amino acid similarities in the large subunit of the ring hydroxylating dioxygenase, which initiates PAH metabolism. Based on those RHD similarities, several groups of PAH-degrading bacteria have been resolved [4, 5], and *D. acidovorans* Cs1-4 is the first representative of the group designated as the Phn family to have a full genome sequence determined. A draft genome sequence has also been determined for a second representative of the Phn family, *Burkholderia* sp. Ch1-1 (GenBank ADNR00000000; [6]).

Bacteria belonging to different RHD groups typically differ in other characteristics relevant to PAH metabolism including the range of PAH degraded, pathways for PAH metabolism and the structure of gene clusters encoding PAH degradation [7]. Furthermore, lateral transfer of RHD genes of different phylogenetic groups appears to be mediated by different types of mobile genetic elements [8]. The full genome sequence of *D. acidovorans* Cs1-4 can thus provide insights into a variety of aspects of PAH metabolism in general, and phenanthrene degradation in particular.

## Organism information

### Classification and features

The genus *Delftia* is a phylogenetically cohesive group within the *Comamonadaceae* family of the *Betaproteobacteria* (Table 1, Fig. 2). At the time of writing, permanent draft genome sequences were publically available for four draft or finished genome sequences of *D. acidovorans* (including *D. acidovorans* Cs1-4)*.* But, of those four genomes, only those of strains Cs1-4 and SPH1 (NC_010002) appear to be *bone fide* representatives of this species, as 16S rRNA sequences of the other two strains, CCUG 247B and CCUG 15835, best match to *Delftia tsuruhatensis* (Table 2). These species affiliations were supported by a phylogenetic tree of the *Comamonadaceae*, which resolved three species clusters within *Delftia*: *D. acidovorans*, *D. tsruhatensis* and *D. litopenaei* (Fig. 2). Strains CCUG 247B and CCUG 15835 clustered with *D. tsruhatensis* instead of *D. acidovorans**.* A fourth species, *D. lacustris*, clustered with *D. tsruhatensis* and thus, in this analysis, did not have strong phylogenetic support as a distinct species (Fig. 2).

**Table 1:** Classification and general features of *Delftia acidovorans* Cs1-4 according to MIGS recommendation [20]

MIGS ID	Property	Term	Evidence code^a^
	Current classification	Domain *Bacteria*	TAS [26]
		Phylum *Proteobacteria*	TAS [27]
		Class *Betaproteobacteria*	TAS [28, 29]
		Order *Burkholderiales*	TAS [30, 29]
		Family *Comamonadaceae*	TAS [31]
		Genus *Delftia*	TAS [32]
		Species *acidovorans*	TAS [32]
	Gram stain	Negative	TAS [2]
	Cell shape	Rod	TAS [2]
	Motility	Motile	TAS [2]
	Sporulation	Nonsporulating	TAS [2]
	Temperature range	Mesophilic	TAS [2]
	Optimum temperature	30 °C	TAS [2]
	pH range, optimum	5.5-7.5, 7.0	TAS [2]
	Carbon source	Phenanthrene, pyruvate, vanillate, succinate, Formic acid, gluconic acid, malonic acid, hydroxybutyric acid, lactic acid, propionic acid	TAS [2]
MIGS-6	Habitat	Oil fields, sludge, freshwater, soil	TAS [2]
MIGS-6.3	Salinity	Fresh water only	TAS [2]
MIGS-22	Oxygen requirement	Aerobic	TAS [2]
MIGS-15	Biotic relationship	Free-living	TAS [2]
MIGS-14	Pathogenicity	None	TAS [2]
MIGS-4	Geographic location	Chippewa Falls, Wisconsin, USA	TAS [2]
MIGS-5	Sample collection	2003	TAS [2]
MIGS-4.1	Latitude	44.9369 °N	TAS [1]
MIGS-4.2	Longitude	91.3928 °W	TAS [1]
MIGS-4.4	Altitude	256 m	TAS [1]

^a^Evidence codes - IDA: Inferred from Direct Assay; TAS: Traceable Author Statement (i.e., a direct report exists in the literature); NAS: Non-traceable Author Statement (i.e., not directly observed for the living, isolated sample, but based on a generally accepted property for the species, or anecdotal evidence). These evidence codes are from the Gene Ontology project [33]

**Table 2:** Summary of currently available genome sequence data from bacteria identified as *Delftia acidovorans*

Genome Name	Taxon OID	Status	16S rRNA Gene ID^a^	16S rRNA Size (bp)	BLAST-N Match	Identity	Accession Number of BLAST-N Match
*Delftia acidovorans* Cs1-4^b^	650716032	Finished	650858110	1521	*Delftia acidovorans* Cs1-4	100 %	NR_074626
*Delftia acidovorans* SPH-1	641228489	Finished	641295730	1526	*Delftia acidovorans* SPH-1	100 %	NR_074691
*Delftia acidovorans* CCUG 15835	2541046983	Draft	2541343813	1521	*Delftia tsuruhatensis*ARB-1	100 %	KC572558
*Delftia acidovorans* CCUG 274B	2541046984	Draft	2541347625	1521	*Delftia tsuruhatensis*ARB-1	100 %	KC572558

^a^Gene ID is as assigned by the JGI Integrated Microbial Genomes database

^b^Genome sequence determined under the name *Delftia* sp. Cs1-4

*Delftia acidovorans* Cs1-4 was isolated from soil contaminated by coal tar at the former site of a manufactured gas plant in Chippewa Falls, WI by using an enrichment culture with phenanthrene as the sole added carbon source [1]. Strain Cs1-4 has since been used as the model organism for the study of nanopods [2, 3]. *Delftia acidovorans* Cs1-4 is deposited in the culture collection of the United States Department of Agriculture, Agricultural Research Service (Peoria, IL) as strain NRRL B-65277.

**Fig. 3:**
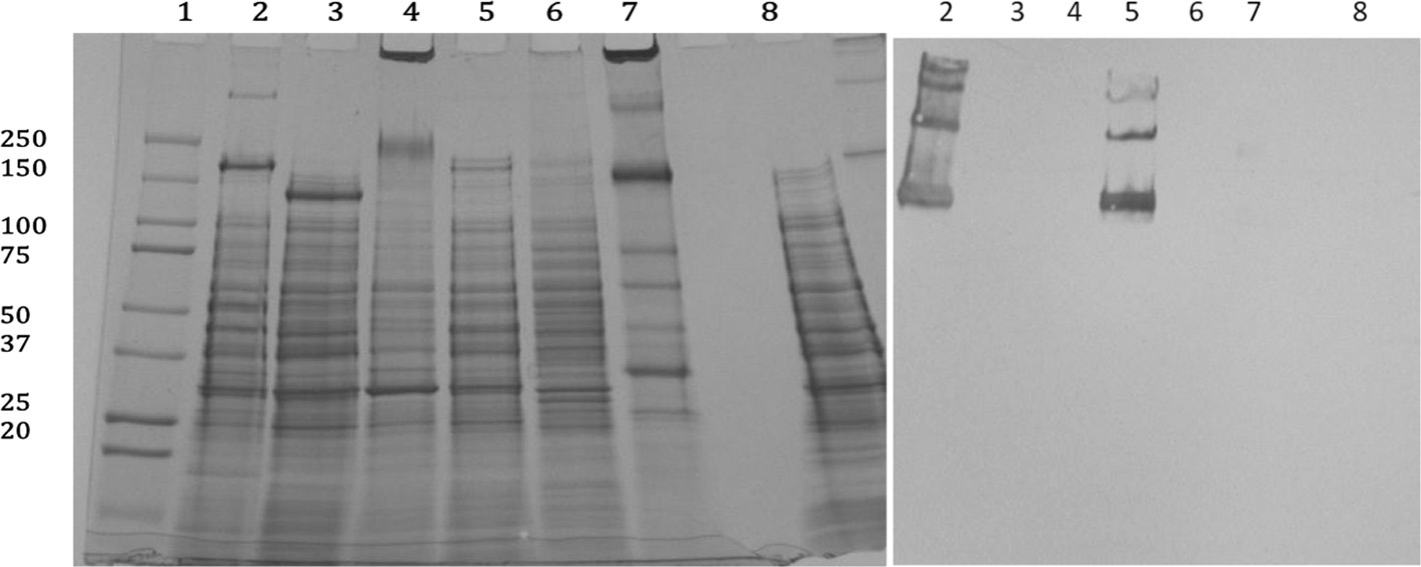
Antigenic relation of NpdA from *Delftia acidovorans* Cs1-4 with other members of *Comamonadaceae* family. (Left panel) SDS PAGE . (right panel) Immunoblot with anti-NpdA antibody. Lane 1, Molecular weight marker; lane 2, *Delftia acidovorans* Cs1-4 (phenanthrene-grown); lane 3, *Delftia acidovorans* SPH1; lane 4, *Deftia acidovorans* ATCC15688; lane 5, *Delftia acidovorans* Cs1-4 (nutrient broth-grown); lane 6, *Acidovorax delafieldii* 2AN; lane 7, *Verminephrobacter eisenae* EF01-2; Lane 8, M3. Each lane was loaded with 50 μg of protein, and the gel stained with Coomasie blue. Preparation of anti-NpdA IgY and immunoblotting procedures are described in Shetty et al. [2]

The phospholipid fatty acid profile of *D. acidovorans* Cs1-4 (cells grown on Tryptic Soy Broth and PLFA prepared by the Bligh and Dyer method, [9]) was 19:0 CYCLO ω8c, 2 %; 18:1 ω7c, 6 %; 18:0, 9 %; 17:0 CYCLO, 23 %; 16:1 ω7c, 11 %; 16:0, 36 %; sum of minor PLFA, 14 %. The major quinone is ubiquinone. The glycosyl composition of lipopolysaccharide was (mole %): rhamnose, 49.1 %, glucose, 38.0 %, N-acetylglucosamine, 10.7 % and N-acetylgalactosemine, 2.2 %. The LPS was prepared by a Tri reagent method [10] and monosaccaride content determined by liquid chromatography-mass spectrometry. A distinguishing characteristic of *D. acidovorans* is a novel surface layer protein, NpdA [2] and NpdA of *D. acidocvorans* Cs1-4 was antigenically distinct form that of *D. acidovorans* 15668 and SPH1 (Fig. 3). Immunoblotting was done following the method of Gallager et al. 2001 [11] with anti-NpdA prepared as described by Shetty et al. 2011 [2].

Biolog GN plates (Biolog, Hayward, CA) were used to assay metabolic characteristics of *D. acidovorans* Cs1-4, as well as *D. acidovorans* strains SPH1 and ATCC 15668. All three were positive for metabolism of: Tween 80, Tween 40, D-mannitol, D-psicose, pyruvic acid methyl ester, succinic acid mono-methyl-ester, *cis*-aconitic acid, formic acid, D-gluconic acid, α-hydroxybutyric acid, β-hydroxybutyric, γ-hydroxybutyric acid, β-hydroxyphenylacetic acid, itaconic acid, α-ketobutyric acid, α-ketoglutaric acid, α-ketovaleric acid, L-lactic acid, malonic acid, propionic acid, quinic acid, D-saccharic acid, sebacic acid, succinic acid, bromosuccinic acid, L-alaninamide, D-alanine, L-alanine, L-alanylglycine, L-aspartic acid, L-histidine, L-leucine, L-phenylalanine, L-proline, L-pyroglutamic acid, L-threonine, γ-amino butyric acid, urocanic acid and glycerol.

Strains Cs1-4, SPH1 and ATCC 15668 were negative for metabolism of: α-cyclodextrin, dextrin, glycogen, *N*-acetyl-D-galactosamine, *N*-acetyl-D-glucosamine, adonitol, L-arabinose, D-arabitol, D-cellobiose, L-fructose, D-galactose, gentiobiose, α-D-glucose, α-D-lactose, lactulose, maltose, D-mannose, D-melibiose, β-methyl-D-glucoside, D-raffinose, L-rhamnose, D-sorbitol, sucrose, D-trehalose, turanose, xylitol, D-galactonic acid lactone, D-galacturonic acid, D-glucosaminic acid, D-glucuronic acid, glycyl-L-aspartic acid, glycyl-L-glutamic acid, hydroxyl-L-proline, L-ornithine, D-serine, L-serine, D-melibiose, L-carnitine, inosine, uridine, thymidine, phenyethylamine, putrescine, 2-aminoethanol, 2,3-butanediol, L-α-glycerol phosphate, α-D-glucose-1-phosphate and D-glucose-6-phosphate.

*Delftia acidovorans**s*trains Cs1-4, SPH1 and ATCC 15668varied in metabolism of six substrates: *i*-erythritol, D-fructose, *m*-inositol, citric acid, glucuronamide and L-asparagine. Strain Cs1-4 was positive for only *i*-erythritol, strain ATCC 15688 was positive for D-fructose and citric acid, while strain SPH1 metabolized all six of these compounds.

## Genome sequencing and annotation

### Genome project history

The genome sequence was completed in May 2011 and presented for public access on December 2011 and is available in GenBank (NC_015563). Quality assurance of the genomic DNA preparation used for sequencing was done in the laboratory of W.J. Hickey at the University of Wisconsin-Madison. Sequencing, finishing and annotation were performed by the U.S. DOE JGI. A summary of the project information is shown in Table 3.

**Table 3:** Project information

MIGS ID	Property	Term
MIGS-31	Finishing quality	Level 6 Finished
MIGS-28	Libraries used	Titanium draft library, paired end library and Illumina library
MIGS-29	Sequencing platforms	454 Titanium, Illumina
MIGS-31.2	Fold coverage	38.4 × 454 Titanium, 89.4 × Illumina
MIGS-30	Assemblers	Newbler, Velvet
MIGS-32	Gene calling method	Prodigal, GenePRIMP
	Locus tag	DelCs14
	Genbank ID	NC_015563
	Genbank Date of Release	December 1, 2011
	GOLD ID Bioproject	Gc0178467319 67319
MIGS-13	Source Material Identifier	Cs1-4
	Project relevance	Biotechnological, Environmental

### Growth conditions and genomic DNA preparation

*Delftia acidovorans* Cs1-4 was grown aerobically in Nutrient Broth at 30 °C. DNA was isolated from 100 mL of overnight culture by a CTAB method [12]. Cells were collected by centrifugation (10,000 × *g* 10 min) and then resuspended in Tris-EDTA buffer to OD600 of 1.0. Lysozyme (100 mg/mL) was added followed by 10 % SDS and proteinase K (10 mg/mL). After incubation for 1 h at 37 °C, 5 M NaCl and CTAB/NaCl were added, and the solution incubated at 65 °C for 10 min. DNA was purified by phenol chloroform extraction, and then re-suspended in TE buffer with RNase (10 mg/mL). For quality confirmation, the DNA preparation was run on a 1 % agarose gel with phage λ DNA as a mass standard (DOE JGI).

### Genome sequencing and assembly

The draft genome of *D. acidovorans* Cs1-4 was generated at the DOE JGI using a combination of Illumina and 454 technologies. An Illumina GAii shotgun library which generated 16,416,493 reads totaling 591 Mb, a 454 Titanium standard library which generated 770,171 reads and 1 paired end 454 library with an average insert size of 8 kb which generated 508,092 reads totaling 372.8 Mb of 454 data, was constructed and sequenced. The initial draft assembly contained 40 contigs in 2 scaffolds. The 454 Titanium standard data and the 454 paired end data were assembled together with Newbler, version 2.3. The Newbler consensus sequences were computationally shredded into 2 kb overlapping shreds. Illumina sequencing data was assembled with VELVET, version 0.7.63, and the consensus sequence was computationally shredded into 1.5 kb overlapping shreds. We integrated the 454 Newbler consensus shreds, the Illumina VELVET consensus shreds and the read pairs in the 454 paired end library using parallel phrap, version SPS - 4.24 (High Performance Software, LLC). The software Consed was used in the following finishing process. Illumina data was used to correct potential base errors and increase consensus quality using the software Polisher developed at JGI (Alla Lapidus, unpublished). Possible mis-assemblies were corrected using gapResolution (Cliff Han, unpublished), Dupfinisher, or sequencing cloned bridging PCR fragments with subcloning. Gaps between contigs were closed by editing in Consed, by PCR and by Bubble PCR primer walks (J-F Cheng, unpublished). A total of 182 additional reactions were necessary to close gaps and to raise the quality of the finished sequence. The total size of the genome is 6,685,842 bp and the final assembly is based on 253.3 Mb of 454 draft data which provides an average 38.4× coverage of the genome and 590.2 Mb of Illumina draft data which provides an average 89.4× coverage of the genome.

### Genome annotation

Genes were identified using Prodigal as part of the Oak Ridge National Laboratory genome annotation pipeline, followed by a round of manual curation using the JGI GenePRIMP pipeline. The predicted CDSs were translated and used to search the National Center for Biotechnology Information non-redundant database, UniProt, TIGRFam, Pfam, PRIAM, KEGG, COG, and InterPro databases. These data sources were combined to assert a product description for each predicted protein. Non-coding genes and miscellaneous features were predicted using tRNAscan-SE, RNAMMer, Rfam, TMHMM, and signalP. The final genome sequence is deposited in GenBank under accession NC_015563.

## Genome properties

**Fig. 4:**
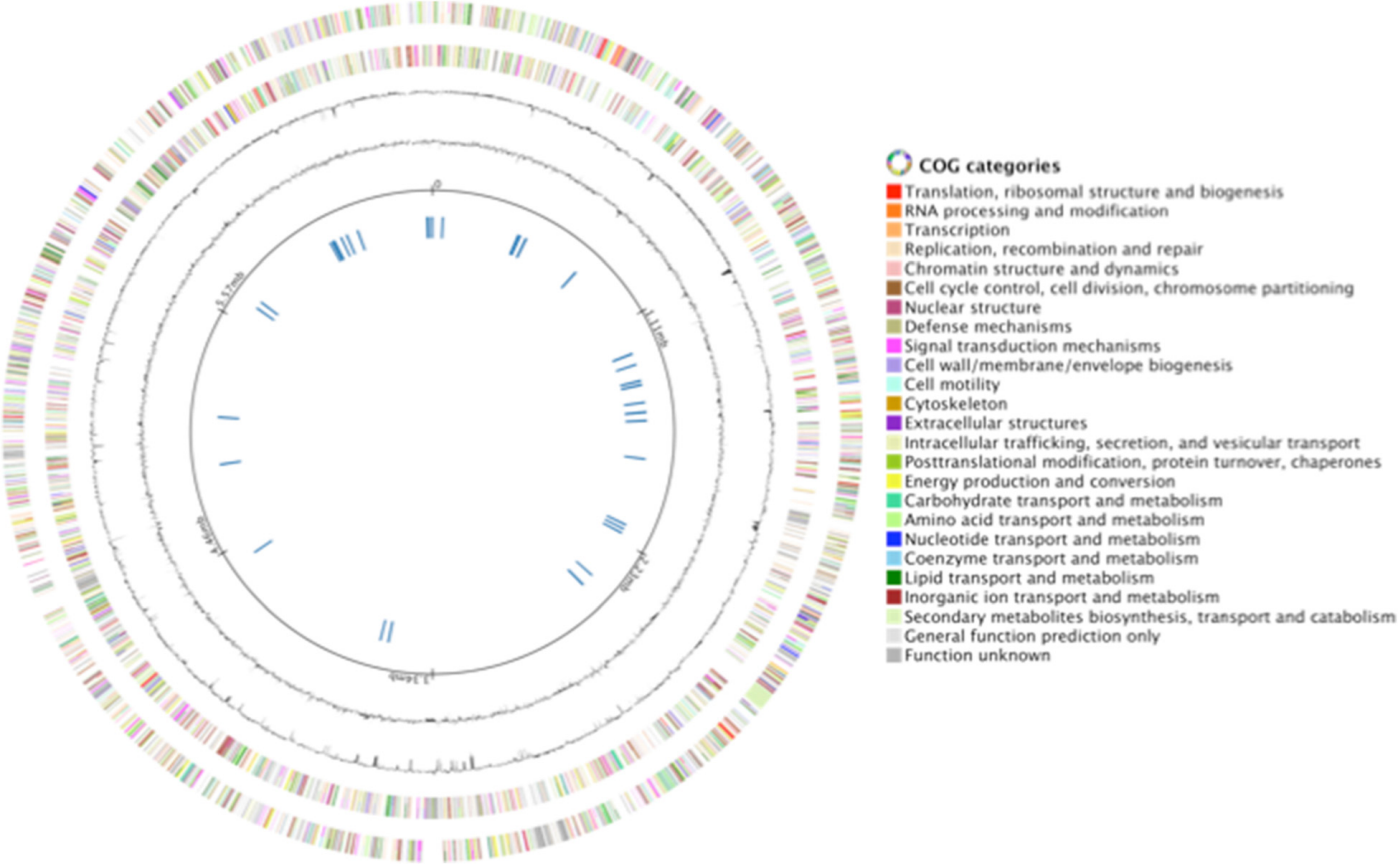
Circular representation of the *Delftia acidovorans* Cs1-4 chromosome.Features displayed are (outside to center): forward strand genes (colored by COG categories), reverse strand genes (colored by COG categories), RNA genes (tRNA, green; rRNA, red; other RNA, black), GC content and GC skew.

The genome of strain Cs1-4 consists of a single circular chromosome of 6,685,842 bp (66.7 % G + C content) containing with 6,028 predicted genes (Fig. 4, Table 4). There were 5,931 protein-encoding genes; 4,425 of these gene products were assigned to a putative function with the remaining annotated as hypothetical proteins (Table 5). There were 80 tRNA genes and five 16S rRNA genes. For the latter, three were 100 % identical to each other, while two were 99 % identical (DelCs14_R0076, DelCs14_R0098).

**Table 4:** *Delftia acidovorans* Cs1-4 genome statistics

Attribute	Value	% of Total
Genome size (bp)	6,685,842	100
DNA coding (bp)	5,998,883	89.73
DNA G + C content (bp)	4,460,657	66.72
DNA scaffold	1	1.00
Total genes	6,028	100.00
Protein coding genes	5,931	98.39
RNA genes	97	16.1
Pseudo genes	70	1.16
Genes in internal clusters	857	14.22
Genes with function prediction	4,425	73.41
Genes assigned to COGs	4,845	80.37
Genes assigned to Pfam domains	5,061	83.96
Genes with signal peptides	2,505	41.56
Genes with transmembrane helices	1,377	22.84
CRISPR repeats	3	NA

**Table 5:** Number of genes in *Delftia acidovorans* Cs1-4 associated with the 25 general COG functional categories

Code	Value	% of total^a^	Description
J	207	3.78	Translation
A	2	0.04	RNA processing and modification
K	581	10.62	Transcription
L	168	3.07	Replication, recombination and repair
B	2	0.04	Chromatin structure and dynamics
D	32	0.58	Cell cycle control, mitosis and meiosis
V	71	1.3	Defense mechanisms
T	362	6.62	Signal transduction mechanisms
M	251	4.59	Cell wall/membrane biogenesis
N	138	2.52	Cell motility
U	145	2.65	Intracellular trafficking and secretion
O	162	2.96	Posttranslational modification, protein turnover, chaperones
C	346	6.32	Energy production and conversion
G	242	4.42	Carbohydrate transport and metabolism
E	479	8.76	Amino acid transport and metabolism
F	88	1.61	Nucleotide transport and metabolism
H	191	3.49	Coenzyme transport and metabolism
I	261	4.77	Lipid transport and metabolism
P	368	6.73	Inorganic ion transport and metabolism
Q	172	3.14	Secondary metabolites biosynthesis, transport and catabolism
R	621	11.35	General function prediction only
S	580	10.60	Function unknown
-	1183	19.63	Not in COGs

The total is based on the total number of protein coding genes in the annotated genome

## Insights from the genome sequence

### Comparisons with other sequenced *Delftia* genomes

The genome of strain Cs1-4 was similar to that of the other available *Delftia* genomes with respect to categories such as gene count and % G + C (Table 4). However, there was a clear distinction between these strains in the area of ribosomal genes, as their abundance was nearly identical in the genomes of strains Cs1-4 and SPH1, but much greater than that in strains CCUG 274B and CCUG 15835. There was also a marked difference between the organisms in the area of function prediction, with strains Cs1-4 and SPH1 having a much larger portion of genes with function predicted than did strains CCUG 274B and CCUG 15835. This divergence of the latter two strains from strains Cs1-4 and SPH1 would further support re-assignment of strains CCUG 274B and CCUG 15835 to a *Delftia* species other than *acidovorans* (*e.g.*, *D. tsruhatensis*).

**Fig. 5:**
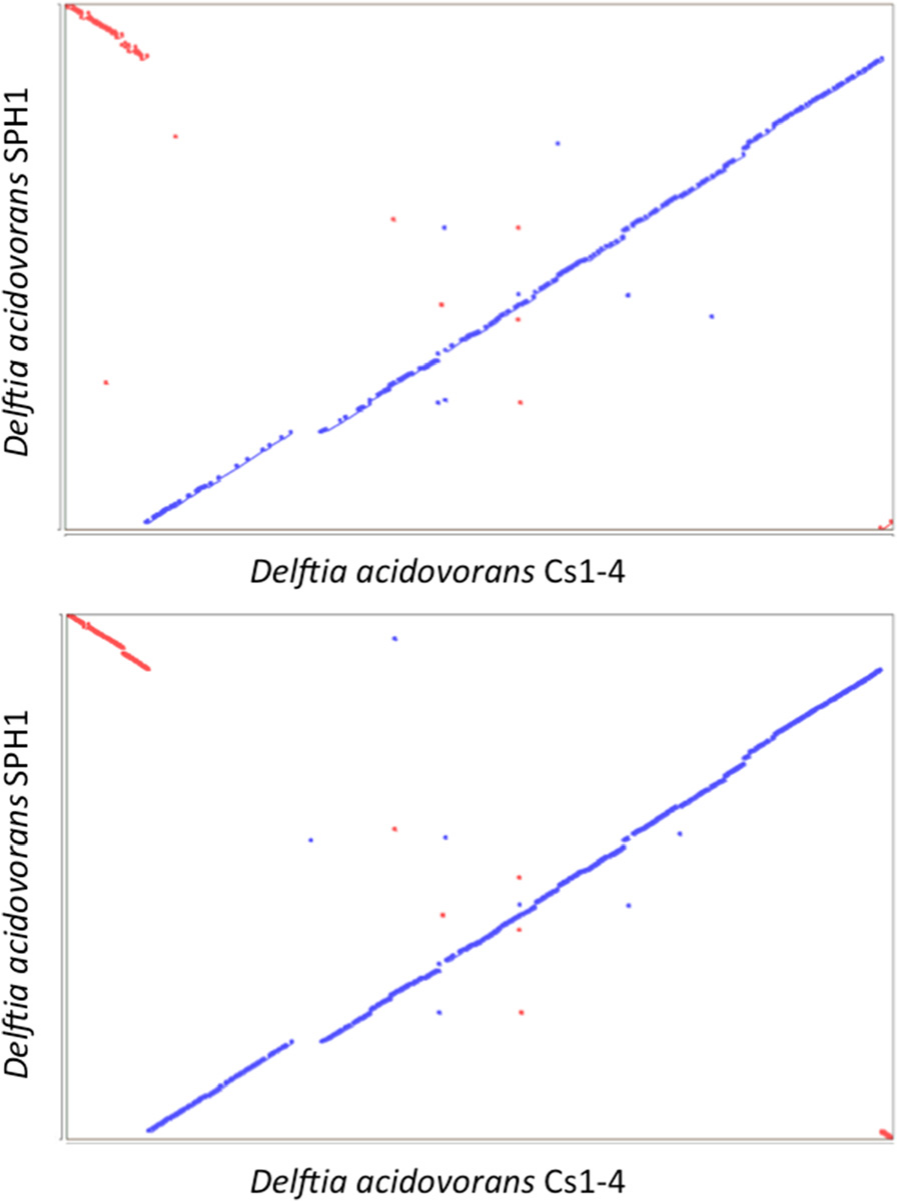
Dot plots of nucloetide (top panel) and protein (bottom panel) between *D. acidovorans* Cs1-4 and *D. acidovorans* SPH1

The closest relative of *D. acidovorans* Cs1-4 with genome sequence data is *D. acidovorans*SPH1. Strain SPH1 was isolated from a sewage treatment plant in Germany as a part of a microbial consortium that degraded linear alkylbenzene sulfonates [13]. Strains Cs1-4 and SPH1 had 99 % identity over the full length of the 16S rRNA gene. Compared to the type strain *D. acidovorans*ATCC 15668, both strains Cs1-4 and SPH1 had 99 % identity over 99 % of the 16S rRNA gene. The genomes of strain Cs1-4 and strain SPH1 had an average nucleotide identity (ANI) of 97.48 %, which qualified the strains as the same species using the 95 % ANI threshold [14, 15]. Based on this high level of nucleotide sequence similarity, a common species is reasonable for these bacteria. But, while the genomic composition of strains SPH1 and Cs1-4 was similar, dot plots exhibited an “X” alignment (Fig. 5), which is indicative of inversions about the origin of replication [16].

Alignment of the *D. acidovorans* Cs1-4 and SPH1 genomes revealed in the former a large genomic island (232 kb) termed the *phn* island that was absent from strain SPH1 [6]. The island contained near its 3’ end a bacteriophage P4-like integrase, a type of enzyme often associated with chromosomal integration of mobile genetic elements [17]. Numerous close orthologs of the strain Cs1-4 integrase can be identified by BLAST-P searches of Genbank, and these may serve as starting points for the elucidation of mobile genetic elements possibly related to the *phn* island.

Conversely, genomic alignment of strains Cs1-4 and SPH1 revealed in strain SPH1 a mobile genetic element of *ca.* 67 kb, which was absent from strain Cs1-4. The loci bounding this element in strain SPH1 were (gene name, equivalent loci in strain Cs1-4) Daci_1694 (*rpoH*, Delcs14_4885) and Daci_1756 (*inclR*, Delcs14_4884). The mobile genetic element in strain SPH1 contained metal detoxification functions, and had extensive similarity to a region in the genomes of strains CCUG 274B and CCUG 15835.

The secretory machinery of strain Cs1-4 were similar to strain SPH1, as well as to strains CCUG 274B and CCUG 15835. These consisted of Type II, Type IV and Type VI secretion system (T6SS) along with the components of a Sec-Signal Recognition Particle Translocon and the *tatABC* genes of the Twin-Arginine Translocation pathway. For all strains, there was a single T6SS cluster. The functions of T6SS have been explored mostly in pathogenic bacteria, where a common feature is mediation of intercellular contact in antagonistic interactions [18]. Functions of T6SS in environmental bacteria such as the *D. acidovorans* strains discussed here are unknown.

*Delftia acidovorans* Cs1-4 produces a novel surface layer protein, which is essential for the formation of extracellular structure called nanopods [2]. The surface layer protein (termed Nanopod Protein A, NpdA) is encoded by DelCs14_5206 (*npdA*), and orthologs of *npdA* are present in the genomes of *D. acidovorans* SPH1 as well as *D. acidovorans* strains CCUG 274B and CCUG 15835.

### Profiles of metabolic networks and pathways

#### Characterization of phenanthrene catabolism genes

Genes for the entire phenanthrene catabolic pathway were identified on a novel 232 kb genomic island named the *phn* island [6] and were segregated into three clusters that were predicted to encode the metabolism of phenanthrene to *ortho*-phthalate (*phn* genes), *ortho*-phthalate to protocatechuate (*oph* genes) and *meta*-cleavage of protocatechuate to pyruvate and oxaloacetate (*pmd* genes). These clusters were non-contiguous; the *oph* and *pmd* clusters were separated from each other by *ca*. 5 kb and located 60 kb upstream of the *phn* cluster. The *phn* gene cluster had a %G + C content of *ca.* 50, which differed significantly from the 66.7 % G + C content of the Cs1-4 chromosome, while %G + C of the *oph* and *pmd* genes was similar to the chromosomal backbone. The G + C content of *Comamonadacea* genomes ranges from 60 % to 70 %, thus the phylogenetic origin of the *phn* genes is outside of this family and likely outside of the order *Burkholderiales*.

#### Styrene degradation via the phenyl acetate pathway

Styrene is often a soil pollutant and strain Cs1-4 possessed genes predicted to encode its degradation by the phenylacetate pathway. The putative styrene operon (conversion of styrene to phenyl acetate [19]), consisted of a regulatory element (*marR*, DelCs14_4846), dienelactone hydrolase (DelCs14_4847), flavin reductase (*styB*, DelCs14_4848), monooxygense (*styA*, DelCs14_48449), short chain dehydrogenase, (DelCs14_4850) and AraC-type transcriptional regulator (DelCs14_4851).

The genetics of phenyl acetate metabolism has been studied in a variety of bacteria, and thirteen genes encoding its transformation to succinyl-CoA and acetyl-CoA have been identified [20, 21]. These genes often occur in a single cluster, but in strain Cs1-4, they are dispersed across the genome. The single largest cluster contained *paaABCDE* (DelCs14_5720-24) and *paaK* (DelCs14_5725), which were predicted to encode a phenylacetyl-CoA epoxidase and phenylacetate-CoA ligase, respectively. Other orthologs that were identified included an epoxyphenylacetyl-CoA isomerase (*paaG*, DelCs14_0512) and a ring-opening enzyme (*paaN*, DelCs14_5726).

#### Benzoate degradation by the benzoyl-CoA pathway

Metabolism of benzoate is important in the degradation of many aromatic compounds, and benzoate degradation by aerobic bacteria is most commonly initiated by oxygenases. In contrast, growth of strain Cs1-4 on benzoate was predicted to proceed by an alternative pathway in which benzoyl-CoA is formed as the primary metabolite [22]. The gene cluster putatively conferring this function included an ABC-type transporter (DelCs14_0078-82), a benzoate-CoA ligase (DelCs14_0073) and a benzoate oxygenase (*boxABC*, DelCs14_0073-0075).

#### Nicotinic acid metabolism by the maleamate pathway

Biodegradation of nicotinic acid has been widely studied as a model for metabolism of *N*-heterocycles. Also, there is interest in the application of bioremediation of nicotine-contaminated soils at tobacco processing facilities. Jime'nez et al. 2008 [23] conducted a detailed analysis of genes in *Pseudomonas putida* KT2440 encoding nicotinic degradation by the maleamate pathway, and strain Cs1-4 possesed a similar set of genes. The *nic* operon in strain Cs1-4 included a Nic regulator (*marR*, DelCs14_4781), an MFS transporter (*nicT*, DelCs14_4782), nicotinate dehydrogenase subunit B (*nicB2,* DelCs14_4783), nicotinate dehydrogenase subunit A (*nicA*, DelCs14_47840), salicylate 1-monooxygenase (*nicC*, DelCs14_4785), maleate isomerase (*nicE*, DelCs14_4786), *N*-formylmaleamate deformylase (*nicD*, DelCs14_4787), 2,5-dihydroxypyridine 5,6-dioxygenase (*nicX*, DelCs14_4788), maleamate amidohydrolase (*nicF,* DelCs14_4789) and an aldehyde dehydrogenase (*nicB1*, DelCs14_4790).

#### Pesticide degradation: Dicamba and Fenitrothion

Two gene clusters in the genome of strain Cs1-4 resembled clusters identified in other proteobacteria that coded for the metabolism of Dicamba (2-methoxy-3,6-dichlorobenzoate) or Fenitrothion (*O*,*O*-dimethyl *O*-[3-methyl-4-nitrophenyl] phosphorothioate). The Dicamba cluster in strain Cs1-4 encompassed a *0*-demethylase that was composed of an oxygenase (DelCs14_5158), ferredoxin (DelCs14_5157) and reductase (DelCs14_5158), and the predicted product of this cluster would be 3,6-dichlorosalicylate [24]. The pathway for 3,6-dichlorosalicylate metabolism is not well delineated, but strain Cs1-4 has putative salicylate oxygenases that could mediate ring hydroxylation (*e.g.*, products of DelCs14_3111, DelCs14_3828).

For fenitrothion, biodegradation could occur via methyl hydroquinone as an intermediate, as it possessed a cluster of genes with similarity to those encoding fenitrothion metabolism in *Burkholderia* sp. NF100 [25]. These genes putatively encoded were a flavoprotein monooxygenase (*mhqA*, DelCs14_5563), extradiol dioxygenase (*mhqB*, DelCs14_5562), a ring hydroxylating oxygenase (DelCs14_5559), ferredoxin (DelCs14_5558) and hydrolase (*mhqA*, DelCs14_5554).

## Conclusions

Determination of the complete genome sequence of *D. acidovorans* Cs1-4 achieved the objective of enabling new insights into the genes underlying PAH metabolism and evolutionary mechanisms that may shape the process in the environment. Furthermore, the genome sequence data suggested that biodegradative capacity of *D. acidovorans* Cs1-4 extended beyond PAH, and the organism was well equipped for growth in soils contaminated by a variety of compounds such as *N*-heterocycles and pesticides.

## Abbreviations

PAH: Polycyclic aromatic hydrocarbons
RHD: Ring hydroxylating dioxygenase
ANI: Average nucleotide identity
NpdA: Nanopod protein A
DOE JGI: Department of Energy Joint Genome Institute
T6SS: Type VI secretion system
LPS: Lipopolysaccaride
PLFA: Phospholipid fatty acid
